# Evaluating the Reliability of Anatomic Landmarks in Safe Lumbar Puncture Using Magnetic Resonance Imaging: Does Sex Matter?

**DOI:** 10.1155/2011/868632

**Published:** 2011-06-28

**Authors:** Maryam Rahmani, Seyed Mehran Vaziri Bozorg, Ahmad Reza Ghasemi Esfe, Afsaneh Morteza, Omid Khalilzadeh, Elham Pedarzadeh, Madjid Shakiba

**Affiliations:** ^1^Advanced Diagnostic & Interventional Radiology Research Center (ADIR), Medical Imaging Center, Imam Khomeini Hospital, Tehran University of Medical Sciencse, Tehran 14176-13151, Iran; ^2^Department of Radiology and Imaging, Imam Khomeini Hospital, Tehran University of Medical Sciences, Keshavarz Boulevard, Tehran 14197-33141, Iran

## Abstract

*Aim*. To determine the level of the conus medullaris-Tuffier's line, and conus medullaris-Tuffier's line distance using imaging and evaluate their relation to age and gender. *Methods*. We performed a cross-sectional study of 189 adult participants, who underwent MR imaging of lumbosacral spine. Each vertebra was divided into 3 equal segments (upper, middle, and lower), and intervertebral disc space was also assumed as one segment. All segments from T12 upper segment to L5S1 intervertebral disc were numbered consecutively. The position of conus medullaris and Tuffier's line was determined by the vertebral segment or intervertebral disc space at the same level. The patients were stratified into high/low conus medullaris position (cutpoint: L1 middle segment) and short/long conus-Tuffier's distance (cutpoint: 14 segments). *Results*. Women with low conus were significantly more than men, in patients older than 50 years old (72.7% in females versus 55.3% in males; *P* < .05), whereas there was not such a sexual dimorphism in patients younger than 50 years old. Similarly, short conus-Tuffier's distance was more frequent among women than men in patients older than 50 years old (59.7% in females versus 39.5% in males; *P* < .05), whereas there was not any gender difference in patients younger than 50 years old. Conus-Tuffier's distance was negatively correlated with age (*r* = −0.32, *P* < .001) in all studied population. *Conclusion*. Anatomical landmarks vary according to age and gender, with a lower end of conus medullaris in women, so clinicians should use more caution on the identification of the appropriate site for lumbar puncture, particularly in elderly women.

## 1. Introduction

Conus medullaris is the cone-shaped terminal part of the spinal cord that is usually located between the 12th thoracic (T12) vertebra and the 3rd lumbar (L3) vertebra. Tuffier's line is a clinical landmark defined as a horizontal line connecting the superior aspect of the posterior iliac crests, used as a reference to localize 4th lumbar (L4) vertebra body before performing a lumbar puncture [1]. 

Previous studies showed that trained anesthesiologists fail to correctly identify the lumbar interspaces through physical exam [2]. Also, MR imaging evidences a variable conus medullaris and Tuffier's line position according to age, gender, and race [3–5]. Misidentification of L4 using Tuffier's line causes conus medullaris iatrogenic trauma during anesthesia and lumbar puncture procedures. Therefore, the correct position of these anatomic landmarks is important to execute this procedure safely [6]. Moreover, there are several reports of damage to conus medullaris by lumbar puncture needle during lumbar anesthesia particularly in women [7, 8]. The purpose of this study is to determine the level of the conus medullaris, Tuffier's line, and conus medullaris-Tuffier's line distance in an adult population with low back pain and its relation to their age and gender.

## 2. Materials and Method

From May 2008 to April 2009, a total of 189 adult patients served by our hospital (between 20 and 73 years old), who underwent lumbosacral spine MR imaging as part of workup for low back pain, were included. Lumbosacral X-ray was performed in all participants before MR imaging. Patients with kyphoscoliosis, history of previous spine surgery, spinal fracture, spinal collapse, congenital spinal anomalies, malignancy, and tethered cord were excluded from this study. The presence of thickened filum terminale was studied using axial images, and, if present, the patients were excluded. All patients were examined using a 1.5 T Signa (General Electric, Signa, USA) superconducting magnet with a 5 × 11 cm surface coil. T1-weighted multiple sagittal images, T2-weighted mid-sagittal, and multiple axial images were routinely obtained in all patients. T1-weighted images were obtained using spin echo (SE). T2-weighted images were obtained using gradient-refocused echo. The section thickness was 4 mm, and interslice gap of sagittal sequences was 1 mm. 

**Figure 1:**
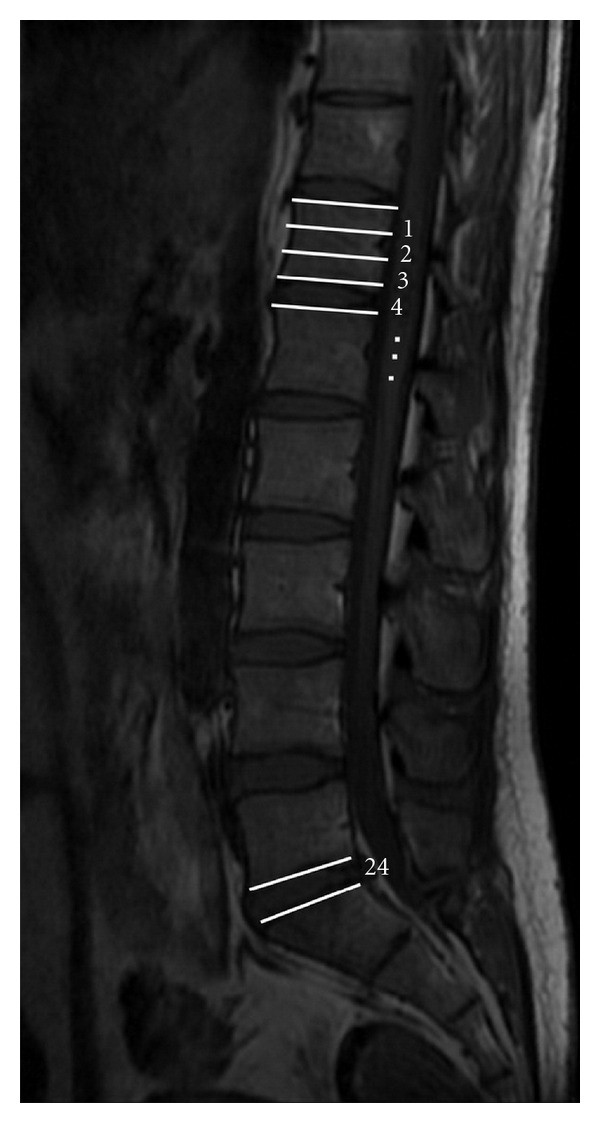
Mid-sagittal T1-weighted MRI of lumbosacral spine. Each vertebra is divided into 3 equal parts of upper, middle, and lower segments. Vertebral segments and disc spaces were numbered consecutively from T12 upper segment downward to L5S1 intervertebral disc.

Each vertebra was divided into 3 equal segments (upper, middle, and lower), and intervertebral disc space was also assumed as one segment. All segments were numbered consecutively from T12 upper segment to L5S1 intervertebral disc (from 1 to 24, resp.) (Figure 1). A horizontal line was drawn from the most distal part of the spinal cord on mid-sagittal MR images perpendicular to the longitudinal axis of the spine. The position of conus medullaris was determined by the corresponding vertebral segment or intervertebral disc space at the same level. Tuffier's line position was determined by a line intersecting the highest part of iliac crests on the anteroposterior (AP) lumbosacral X-ray. The same method used for defining the position of the conus on MRI was used on X-ray images for Tuffier's line. All cases were evaluated by a single radiologist, and the presence of sacralization (a developmental abnormality in which the first sacral vertebra becomes fused with the fifth lumbar vertebra) and lumbarization (nonfusion of the first and second segments of the sacrum so that there is one additional articulated vertebra, the sacrum consisting of one fewer segment) was noted if present.

 Tuffier's line is actually found on physical examination, and we needed to validate our method of determination of Tuffier's line position. We conducted a pilot study on 25 patients after obtaining informed consent. A radiopaque marker was left on the skin on Tuffier's line determined using physical exam. An AP lumbosacral X-ray was performed, and we compared the Tuffier's line determined by the two methods. There was not any significant difference.

**Figure 2:**
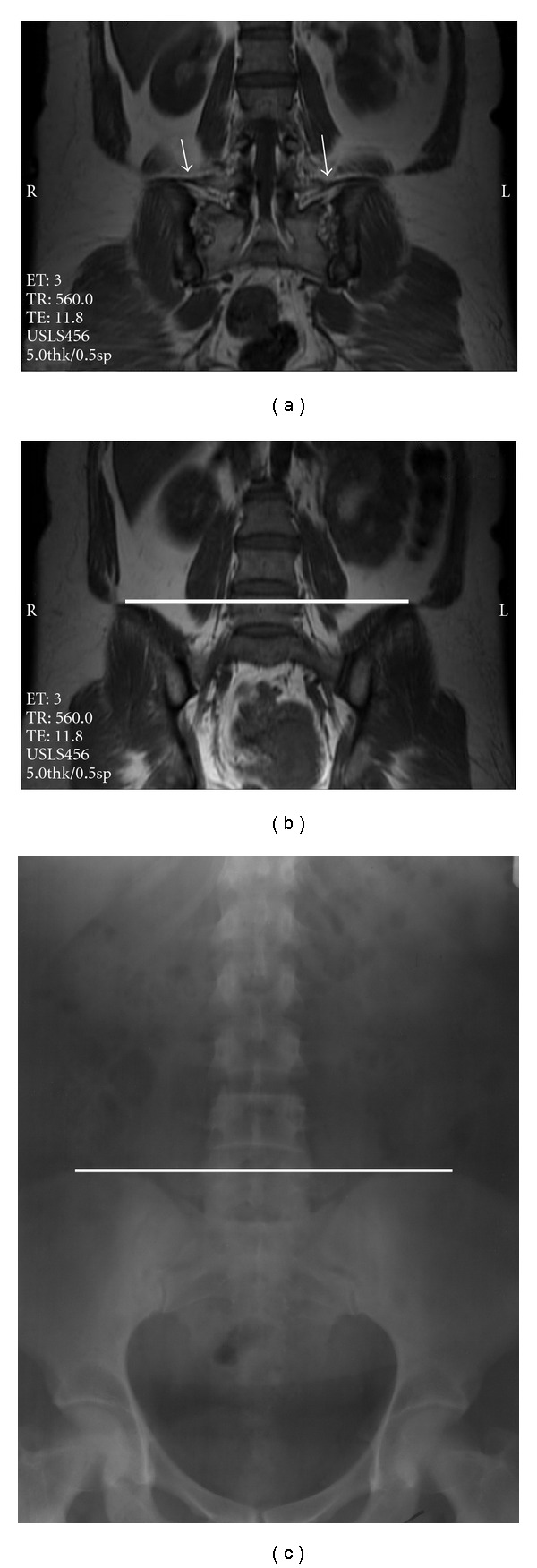
(a) Iliolumbar ligament (arrow) was used to identify L5 vertebra. (b) Coronal MRI showing Tuffier's line at the level of L5 upper segment. (c) Anteroposterior lumbosacral X-ray of the same patient which shows Tuffier's line at the same level.

 On coronal MR images the iliolumbar ligament passes from L5 transverse process to the adjacent part of ilium. That ligament can be used for identification of L5 vertebra, and these images may also be useful for determination of the Tuffier's line position. To assess this possibility, we assumed the line connecting top of iliac crests on coronal MR images which include both iliac crests and vertebral bodies as the Tuffier's line. We compared position of this line with Tuffier's line on lumbosacral X-ray in 30 patients, and we encountered almost the same results (Figure 2).

## 3. Statistical Analysis

The statistical package SPSS 16 for windows (Chicago, Ill, USA) was used for analysis. Kolmogorov-Smirnov test was employed to test the normality of the variables in each group. For comparison of variables between the groups, Mann-Whitney U test (for continuous variables deviated from the normal distribution), independent sample T-test (for normally distributed continuous variables), chi-square test (for categorical variables) were employed. Studies have substantially noted age and sex differences in the position of conus medullaris and Tuffier's line [4]. To study the role of age, the cut-off point of 50 years old was set. To provide a measure of association between conus medullaris position, conus-Tuffier's line distance, age, and sex, the patients were stratified into high conus (conus medullaris position equal and higher than middle L1 vertebra) and low conus (conus medullaris position lower than middle L1 vertebra) as well as short conus-Tuffier's line distance (conus-Tuffier's distance equal and less than 14 segments) and long conus-Tuffier's distance (conus-Tuffier's distance more than 14 segments). The probability of a patient to have a low conus and a short conus-Tuffier's distance according to their age and sex was determined. Pearson correlation was employed to study the correlation between conus-Tuffier's line and age. Significance was set at *P* < .05.

## 4. Results

Table 1 presents the primary characteristics of participants. There were 72 (38.1%) males and 117 (61.9%) females with the mean age of 50.1 and 52.3 years old. The median of conus medullaris position was L1 upper segment (ranging from T12 upper segment to L2 middle segment). The median of Tuffier's line position was in L5 upper segment (ranging from L3L4 intervertebral disc to L5S1 intervertebral disc). The median of conus-Tuffier's line distance was 15 vertebral segments (ranging from 9 to 21 vertebral segments). 

The conus medullaris was significantly lower in females than in males (from T12 upper segment to L2 middle segment (median: L1 middle segment) in females versus from T12 upper segment to L2 upper segment (median: L1 upper segment) in males; *P* < .05) (Table 1). The Tuffier's line was significantly higher in males than in females (between L3L4 intervertebral disc to L5S1 intervertebral disc (median: L4L5 intervertebral disc) in males versus from L4 middle segment to L5S1 intervertebral disc (median: L5 upper segment) in females; *P* < .001). There was not any significant difference in conus-Tuffier's line between males and females (from 9 to 21 (median: 15) in males versus from 10 to 20 (median: 15) in females; *P* = .6). 

There were significantly more women with low conus compared to men in patients older than 50 years old (56/77, 72.7% in females versus 21/38, 55.3% in males; *P* < .05) when there was not such a sexual dimorphism in patients younger than 50 years old (15/40, 37% in females versus 16/34, 47% in males; *P* = .43). Similarly short conus-Tuffier's line distance was more frequent among women than men in patients older than 50 years old (46/77, 59.7% in females versus 15/38, 39.5% in males; *P* < .05) when there was not any gender difference in patients younger than 50 years old (15/40, 37% in females versus 14/34, 41% in males; *P* = .65).

 Conus-Tuffier's line distance was negatively correlated with age (*r* = −0.32, *P* < .001) in our cases. Lumbar spine degenerative joint disease was noted in 80 (42.3%) cases. There was a slight lowering of the conus and decrease in conus-Tuffier's line distance in patients with degenerative joint disease. 

Sacralization was noted in 34 (18%) and lumbarization was noted in 10 (5.3%) of our studied population. Tuffier's line in patients with sacralization (from L5 lower segment to L3L4 intervertebral disc, median: L4L5 intervertebral disc) was upper and in patients with lumbarization (from L5S1 intervertebral disc to L4 middle segment, median: L5 lower segment) was lower than those in healthy volunteers (from L5S1 intervertebral disc to L3L4 intervertebral disc, median: L5 upper segment).

## 5. Discussion

Our findings clearly demonstrated that both conus medullaris and the Tuffier's line were in a significantly lower position in females compared to males. There were a higher number of women with low conus and short conus-Tuffier's distance compared to men in patients older than 50 years old, whereas there was not such a sexual dimorphism in patients younger than 50 years old. 

Both conus medullaris and Tuffier's line have been reported to be placed slightly lower down more frequently in females than in males [9]. To date we are unaware of any study demonstrating gender differences in the conus-Tuffier's line distance. Two other groups, Thomson and Demiryürek et al. showed that conus medullaris is lower in females than in males [10, 11]. Kim and collaborators showed a negative correlation between old age and the position of conus medullaris [4] however, they did not report any sexual dimorphism in old ages [4]. We chose a cutpoint of 50 years old as it is the mean age of menopause in Iran [12]. It is known that postmenopausal women have a higher risk of osteoporosis and spinal fracture compared to men [13–16]. So it was expected to observe a higher frequency of short conus-Tuffier's line among postmenopausal women. Interestingly, we found these results despite excluding patients with spinal fracture and collapsed vertebra, which are usually women [17, 18]. 

On the other hand, women have a higher BMI compared to men. The identification of Tuffier's line requires palpation through a variable amount of subcutaneous fat and would be harder in obese individuals [19]. Hence, we suggest that clinicians should be more cautious in identifying Tuffier's line and appropriate site of puncture in postmenopausal women. 

Our results about the anatomical positions of conus medullaris and Tuffier's line are consistent with previous studies [3, 10]. We showed that there is a safe zone of at least 2 vertebral bodies and disc spaces (9 vertebral segments) between conus medullaris and Tuffier's line. The median of conus medullaris position was L1 upper segment (ranging from T12 upper segment to L2 middle segment). The median of Tuffier's line position was L5 upper segment (ranging from L3L4 intervertebral disc to L5S1 intervertebral disc). Thomson showed that the position of the conus medullaris is from the lower border of T12 and the upper border of L3 [10]. Consistently, Saifuddin et al. showed that the tip of the conus medullaris is between middle segment of T12 and upper segment of L3 with a median position at the lower segment of L1 [3]. In older patients with osteoporosis or age-related vertebral deformity, a reduced height of the vertebral body will be found, and hence Tuffier's line will be higher [20]. We also showed that there is a significant negative correlation between age and conus-Tuffier's line distance, irrespective of gender. 

The principal limitation of the present study is its cross-sectional nature, which precludes the determination of the direction of causality. Furthermore, we used patients with low back pain as our study population. Patients with other conditions or healthy individuals must be studied in another trial. However, we have relatively large sample size and close similarity between groups in most of the potentially confounding variables. In conclusion, we showed that there should be more caution in using the Tuffier's line as a landmark during lumbar puncture in postmenopausal women.

##  Conflict of Interests

The authors declare that there is no conflict of interests.

## Figures and Tables

**Table 1:** Primary characteristics of participants.

	Male (*n* = 72)	Female (*n* = 117)	Total (*n* = 189)
Age (yrs, SD)	50.1 (4.1)	52.3 (5.2)	51.9 (4.8)
Conus-position	L1m* (T12u-L2m)	L1u (T12u-L2u)	L1u (T12u-L2m)
Tuffier's line position	L4L5 IVD** (L3L4 IVD-L5S1 IVD)	L5m (L4m-L5S1 IVD)	L5u (L3L4 IVD-L5S1 IVD)
Conus-Tuffier's distance (no. of segments)	15 (9–21)	15 (10–20)	15 (9–21)
Younger than 50	34	40	74
Low conus	16 (47%)	15 (37%)	31 (41%)
Short conus-Tuffier's distance	14 (41%)	15 (37%)	29 (39%)
Older than 50 years	38	77	115
Low conus	21 (55.3%)*	56 (72.5%)	77 (67%)
Short conus-Tuffier's distance	15 (39.5%)*	46 (59.7%)	61 (53.0%)

Variables are expressed as mean (SD), median (interquartile range) or number and percent. *P < .05,**P < .01 when comparing women with men. U stands for upper segment; m stands for middle segment; l stands for lower segment; IVD stands for intervertebral disc. Low conus: conus medullaris position lower than middle L1 vertebra; short conus-Tuffier's distance: conus-Tuffier's distance equal and less than L4 segments.
